# Supplementary material for: HIVprotI: an integrated web based platform for prediction and design of HIV proteins inhibitors
Source: J Cheminform. 2018 Mar 9;10:12. doi: 10.1186/s13321-018-0266-y (PMC5845081; doi:10.1186/s13321-018-0266-y)
Supplement: Supplementary file 2 — Additional file 2. Source code of HIVProtI web server. [file 13321_2018_266_MOESM2_ESM.zip › HIVprotI_Source-code/hivprotI/weka-3-6-6/doc/weka/classifiers/trees/m5/YongSplitInfo.html]

YongSplitInfo


---


|  |  |  |
| --- | --- | --- |
| **Overview** \| **Package** \| **Class** \| **Tree** \| **Deprecated** \| **Index** \| **Help** \| **Weka's home** | |  |
| **PREV CLASS**   NEXT CLASS | **FRAMES**    **NO FRAMES**     **All Classes** |  |
| SUMMARY: NESTED \| FIELD \| CONSTR \| METHOD | DETAIL: FIELD \| CONSTR \| METHOD |  |


---


## weka.classifiers.trees.m5 Class YongSplitInfo

```
java.lang.Object
  weka.classifiers.trees.m5.YongSplitInfo
```

**All Implemented Interfaces:**: java.io.Serializable, java.lang.Cloneable, SplitEvaluate, RevisionHandler

---

``` public final class YongSplitInfo extends java.lang.Object implements java.lang.Cloneable, java.io.Serializable, SplitEvaluate, RevisionHandler ```

Stores split information.

**Version:**
:   $Revision: 1.4 $

**Author:**
:   Yong Wang (yongwang@cs.waikato.ac.nz), Mark Hall (mhall@cs.waikato.ac.nz)

**See Also:**: Serialized Form

---

**Constructor Summary**

`YongSplitInfo(int low,
int high,
int attr)`
  
          Constructs an object which contains the split information


| **Method Summary** | |
| --- | --- |
| `void` | `attrSplit(int attr, Instances inst)`             Finds the best splitting point for an attribute in the instances |
| `SplitEvaluate` | `copy()`             Makes a copy of this SplitInfo object |
| `java.lang.String` | `getRevision()`             Returns the revision string. |
| `void` | `initialize(int low, int high, int attr)`             Resets the object of split information |
| `double` | `maxImpurity()`             Returns the impurity of this split |
| `int` | `position()`             Returns the position of the split in the sorted values. |
| `int` | `splitAttr()`             Returns the attribute used in this split |
| `double` | `splitValue()`             Returns the split value |
| `java.lang.String` | `toString(Instances inst)`             Converts the spliting information to string |

**Methods inherited from class java.lang.Object**

`equals, getClass, hashCode, notify, notifyAll, toString, wait, wait, wait`

**Constructor Detail**

### YongSplitInfo

```
public YongSplitInfo(int low,
                     int high,
                     int attr)
```

:   Constructs an object which contains the split information

    **Parameters:**: `low` - the index of the first instance: `high` - the index of the last instance: `attr` - an attribute


**Method Detail**

### copy

```
public final SplitEvaluate copy()
                         throws java.lang.Exception
```

:   Makes a copy of this SplitInfo object

    :   **Specified by:**: `copy` in interface `SplitEvaluate`
    :   **Returns:**: a copy of the object **Throws:**: `java.lang.Exception`

---


### initialize

```
public final void initialize(int low,
                             int high,
                             int attr)
```

:   Resets the object of split information

    :   **Parameters:**: `low` - the index of the first instance: `high` - the index of the last instance: `attr` - the attribute

---


### toString

```
public final java.lang.String toString(Instances inst)
```

:   Converts the spliting information to string

    :   **Parameters:**: `inst` - the instances

---


### attrSplit

```
public final void attrSplit(int attr,
                            Instances inst)
                     throws java.lang.Exception
```

:   Finds the best splitting point for an attribute in the instances

    :   **Specified by:**: `attrSplit` in interface `SplitEvaluate`
    :   **Parameters:**: `attr` - the splitting attribute: `inst` - the instances **Throws:**: `java.lang.Exception` - if something goes wrong

---


### maxImpurity

```
public double maxImpurity()
```

:   Returns the impurity of this split

    :   **Specified by:**: `maxImpurity` in interface `SplitEvaluate`
    :   **Returns:**: the impurity of this split

---


### splitAttr

```
public int splitAttr()
```

:   Returns the attribute used in this split

    :   **Specified by:**: `splitAttr` in interface `SplitEvaluate`
    :   **Returns:**: the attribute used in this split

---


### position

```
public int position()
```

:   Returns the position of the split in the sorted values. -1 indicates that
    a split could not be found.

    :   **Specified by:**: `position` in interface `SplitEvaluate`
    :   **Returns:**: an `int` value

---


### splitValue

```
public double splitValue()
```

:   Returns the split value

    :   **Specified by:**: `splitValue` in interface `SplitEvaluate`
    :   **Returns:**: the split value

---


### getRevision

```
public java.lang.String getRevision()
```

:   Returns the revision string.

    :   **Specified by:**: `getRevision` in interface `RevisionHandler`
    :   **Returns:**: the revision


---


|  |  |  |
| --- | --- | --- |
| **Overview** \| **Package** \| **Class** \| **Tree** \| **Deprecated** \| **Index** \| **Help** \| **Weka's home** | |  |
| **PREV CLASS**   NEXT CLASS | **FRAMES**    **NO FRAMES**     **All Classes** |  |
| SUMMARY: NESTED \| FIELD \| CONSTR \| METHOD | DETAIL: FIELD \| CONSTR \| METHOD |  |


---
